# Integrative transcriptomic profiling of ncRNAs and mRNAs in developing mouse lens

**DOI:** 10.3389/fgene.2024.1405715

**Published:** 2024-06-12

**Authors:** Liyun Zhang, Xin Liu, Wei Li, Kaiqing Liu, Jing Zhang, Xinhua Liu, Jiantao Wang

**Affiliations:** ^1^ Department of Ophthalmology, General Hospital of Central Theater Command, Wuhan, China; ^2^ Shenzhen Eye Hospital, Jinan University, Shenzhen Eye Institute, Shenzhen, Guangdong, China; ^3^ Department of Pediatric Respiratory Medicine, Maternal and Child Health Hospital of Hubei Province, Tongji Medical College, Huazhong University of Science and Technology, Wuhan, China; ^4^ The Department of Urology, The Third Affiliated Hospital of Shenzhen University, Shenzhen, Guangdong, China

**Keywords:** lens development, transcriptome, messenger RNA, long non-coding RNAs, circular RNAs, competing endogenous RNA

## Abstract

In recent years, burgeoning research has underscored the pivotal role of non-coding RNA in orchestrating the growth, development, and pathogenesis of various diseases across organisms. However, despite these advances, our understanding of the specific contributions of long non-coding RNAs (lncRNAs) and circular RNAs (circRNAs) to lens development remains notably limited. Clarifying the intricate gene regulatory networks is imperative for unraveling the molecular underpinnings of lens-related disorders. In this study, we aimed to address this gap by conducting a comprehensive analysis of the expression profiles of messenger RNAs (mRNAs), lncRNAs, and circRNAs at critical developmental time points of the mouse lens, encompassing both embryonic (E10.5, E12.5, and E16.5) and postnatal stages (P0.5, P10.5, and P60). Leveraging RNA-sequencing technology, we identified key transcripts pivotal to lens development. Our analysis revealed differentially expressed (DE) mRNAs, lncRNAs, and circRNAs across various developmental stages. Particularly noteworthy, there were 1831 co-differentially expressed (CO-DE) mRNAs, 150 CO-DE lncRNAs, and 13 CO-DE circRNAs identified during embryonic stages. Gene Ontology (GO) enrichment analysis unveiled associations primarily related to lens development, DNA conformational changes, and angiogenesis among DE mRNAs and lncRNAs. Furthermore, employing protein–protein interaction networks, mRNA–lncRNA co-expression networks, and circRNA–microRNA–mRNA networks, we predicted candidate key molecules implicated in lens development. Our findings underscore the pivotal roles of lncRNAs and circRNAs in this process, offering fresh insights into the pathogenesis of lens-related disorders and paving the way for future exploration in this field.

## 1 Introduction

The vertebrate lens is characterized by its symmetrical, transparent, and refractive cells (Greiling and Clark, 2012; Cvekl and Zhang, 2017). Originating from the surface ectoderm, lens formation is initiated through mutual induction between the optic vesicle and surface ectoderm. Initially, ectodermal tissue adjacent to the optic vesicle thickens to form a lens plate, followed by the formation of a lens vesicle through invagination. Subsequently, cells in the anterior wall of the lens vesicle differentiate into a single layer of the vertical lens epithelium, while cells in the posterior wall elongate to form primary lens fibers, arranged akin to concentric circles within the crystalline cavity (Bassnett and Šikić, 2017; Cvekl and Zhang, 2017). The intricate interplay of signaling and regulatory networks guides cell division and the differentiation of common progenitor cells toward lens development (Gunhaga, 2011). A series of signaling pathways, including BMP, FGF–MAPK, and FGF–PI3K, have been shown to participate in lens development (Cvekl and Zhang, 2017).

Non-coding RNAs (ncRNAs), including microRNAs (miRNAs), long non-coding RNAs (lncRNAs), and circular RNAs (circRNAs), constitute the majority of RNA transcripts and play indispensable roles in regulating gene expression at translational and post-translational levels. LncRNAs, in particular, are recognized as critical regulators of cellular differentiation and organogenesis, exerting their functions through various mechanisms, including cis or trans pathways (Schmitz et al., 2016; Statello et al., 2021). Numerous lncRNAs have been identified as contributors to lens development; for instance, the lncRNA ALB regulates autophagy during human lens development (Fu et al., 2017). Additionally, circRNAs have been implicated in lens-related diseases, such as circRNAs HIPK3 and KMT2E have also been linked to lens epithelial cell proliferation and the pathogenesis of diabetic cataracts, respectively (Liu et al., 2018; Fan et al., 2019).

Several previous studies have conducted mRNA, lncRNAs, and miRNAs transcriptional comparisons of the developing murine lens (Khan et al., 2015; Khan et al., 2016; Anand et al., 2018; Zhao et al., 2018). However, comprehensive expression profiling of different developmental stages of the mouse lens remains scarce. Notably, prior investigations primarily focused on mRNA, miRNA, and lncRNA expression profiles during the embryonic stage of lens development or later stages, post-birth. Furthermore, limited sequencing depth has constrained the discovery of numerous key genes involved in lens development. To comprehensively uncover the key regulatory factors involved in lens development, we conducted a comparative analysis of transcriptome data from various developmental time points of the lens, spanning embryonic (E) days 10.5, 12.5, and 16.5 as well as postnatal (P) days 0.5, 10.5, and 60. Through this analysis, we identified key mRNAs, lncRNAs, circRNAs, and miRNAs potentially involved in lens development. Our study offers novel insights into the developmental processes of lenses and provides valuable clues for understanding the pathogenesis of lens-related diseases.

## 2 Materials and methods

### 2.1 Tissue collection

C57BL/6N mice were provided by Zhejiang Vital River Laboratory Animal Technology Co., Ltd. (Zhejiang, China). All animal experiments were conducted strictly following the guidelines of the Association for Research in Vision and Ophthalmology (ARVO) statement for the care and use of animals and were approved by China Technology Industry Holding (Shenzhen) Co., Ltd. (no.20220086). The mouse embryos were graded by specifying the date of vaginal plug detection as embryonic (E) day 0.5. The microanatomy of the crystalline lenses of mice was performed at the embryonic (E10.5, E12.5, and E16.5) and postnatal stages (P0.5, P10.5, and P60). Each lens development stage contained three biological repeats, each of which comprised multiple lenses (E10.5, *n* = 15; E12.5, *n* = 20; E16.5, *n* = 10; P0.5, *n* = 5; P10.5, *n* = 5; and P60, *n* = 5). At the same time, whole mouse embryos at each developmental stage were obtained and photographed under a microscope to record the complete developmental state of the whole body and eyes of the mice.

### 2.2 RNA extraction, library construction, and RNA sequencing

Total RNA of samples was extracted using the TRIzol reagent (Invitrogen). The purity of RNA was measured using the NanoPhotometer spectrophotometer, and the concentration was detected using a Qubit^®^ RNA Assay Kit with a Qubit^®^ 2.0 Fluorometer. RNA integrity was determined using the Bioanalyzer 2100 System. In this study, a total of 18 complementary DNA (cDNA) libraries were constructed using the NEBNext^®^ Ultra™ RNA Library Prep Kit for Illumina^®^. The quality of the cDNA library was evaluated using Qubit 2.0 and quantitative real-time PCR (qRT-PCR). The library was sequenced on an Illumina NovaSeq 6,000 instrument. Transcriptome data were uploaded to the NCBI database (https://account.ncbi.nlm.nih.gov/) with the accession number PRJNA929701.

### 2.3 Quality analysis, mapping, and transcriptome assembly

Clean reads were obtained by removing reads containing adapters, poly-N, and low-quality reads from raw data. The Q20, Q30, and GC contents of the clean data were calculated to assess their quality. Clean reads were mapped to the mouse genome sequence (downloaded from the UCSC genome browser on 05/2019) using HISAT2 software (Kim et al., 2015). The mapped reads of each sample were assembled using StringTie software (Pertea et al., 2015).

### 2.4 LncRNA and circRNA identification

Processes for identifying candidate lncRNAs: (1) transcripts with exons <2 and lengths <200 bp were removed; (2) the known transcripts, including mRNAs and other types of RNAs (tRNA, rRNA, snoRNA, snRNA, pre-miRNA, and pseudogenes), were filtered by comparison with annotation files using gffcompare (Pertea and Pertea, 2020). The qualified lncRNAs were classified as known lncRNAs; and (3) the coding potential of the transcripts was assessed using the coding potential calculator 2, coding–non-coding index, and Pfam (Mistry et al., 2007; Sun et al., 2013; Kang et al., 2017). The rest of transcripts without coding potential were defined as final novel lncRNAs.

The identification of circRNAs was based on a conjoint analysis using CIRI and find_circ software (Memczak et al., 2013; Gao et al., 2015).

### 2.5 Expression analysis

Fragments per kilobase of transcript sequence per Millions base pairs sequenced (FPKM) was used to evaluate the expression levels of mRNAs and lncRNAs. Transcripts per million (TPM) was used to evaluate the circRNA expression levels. edgeR software was used to analyze the significance of gene expression difference (Robinson et al., 2010). Differentially expressed (DE) mRNAs and lncRNAs were defined according to the corrected *p*-value (padj) < 0.05 and log2 (fold change) > 1.0, but the identification of mRNAs was based on their gene expression levels; DE circRNAs were identified based on the criteria of *p*-value <0.05 and log2 (fold change) > 1.0.

### 2.6 Cluster analysis and gene expression pattern analysis

Cluster and gene expression pattern analyses of DE mRNAs, lncRNAs, and circRNAs were performed using the OmicShare tools (www.omicshare.com/tools).

### 2.7 GO enrichment analyses

The biological functions of DE lncRNAs were predicted based on the co-localization and co-expression correlation between lncRNAs and mRNAs. The cis-target mRNAs predicted the co-localization threshold to 100 kb upstream and downstream of lncRNAs, and the trans-target mRNAs were identified based on the co-expression correlation with Pearson correlation coefficient ≥0.9 between lncRNAs and mRNAs. The functions of the DE circRNAs were revealed by functional analysis of their parental genes. The clusterProfiler software package was used for GO functional enrichment analysis (Yu et al., 2012). The GO terms with *p*-value <0.05 were considered significantly enriched.

### 2.8 Construction of the protein–protein interaction network

The protein–protein interaction (PPI) network was built to clarify the interaction relationship of DE mRNAs using the STRING database (https://string-db.org/), and its comprehensive score >0.4 was selected as the acquisition criterion. Cytoscape software (version 3.5.1) was used to visualize the PPI network (Su et al., 2014).

### 2.9 Construction of the mRNA–lncRNA co-expression network

To screen the key lncRNAs involved in lens development, a co-expression network between common differentially expressed (CO-DE) lncRNAs and their target CO-DE mRNAs was constructed using Cytoscape software (version 3.5.1).

### 2.10 Construction of the circRNA–miRNA–mRNA network

miRanda database (version 1.0, analysis parameters: score ≥150, and energy threshold ≤ −10) was used to predict miRNAs that interacted with CO-DE circRNAs (Enright et al., 2003). The intersection of RNAhybrid (analysis parameters: energy threshold ≤ −10 and *p* ≤ 0.05), TargetScan (http://www.targetscan.org, default parameters), and miRanda database was used to predict miRNAs that interacted with CO-DE mRNAs in the embryonic period (Enright et al., 2003; Krüger and Rehmsmeier, 2006; Ma et al., 2023). Finally, an endogenous competitive ceRNA (circRNA–miRNA–mRNA) network was visualized using Cytoscape software (version 3.5.1).

### 2.11 qRT-PCR validation

A total of six mRNAs and six ncRNAs (three lncRNAs and three circRNAs) with differences in at least one control group were randomly selected for qRT-PCR analysis, and the primers used in this study are listed in Supplementary Table S1. Total RNA was extracted from lens tissue at different developmental stages using the TRIzol reagent (Invitrogen, United States), according to the manufacturer’s protocol. Total RNA after the removal of genomic DNA was used to synthesize the first strand cDNAs using the PrimeScript™ Master Mix (TaKaRa, Dalian, China). qRT-PCR was performed using Hieff^®^ qPCR SYBR Green Master Mix (Yeasen, Shanghai, China) on the StepOnePlus™ real-time PCR system (Applied Biosystems, Foster City, CA, United States). The reaction conditions are as follows: denaturation for 5 min at 95°C, 40 cycles consisting of 10 s at 95°C, and 30 s at 60°C. Each qRT-PCR assay was performed in triplicate, and *GAPDH* was used as an internal reference gene. The relative gene expression level was calculated using the 2^−ΔΔCT^ method.

### 2.12 Statistical analysis

Statistical analyses were performed using SPSS v29.0.2 software (SPSS, Inc.). The results are shown as means ± standard deviation. Statistical differences between any two groups were determined using one-way ANOVA. *p*-value <0.05 was determined to be the criterion for statistically significant differences.

## 3 Results

### 3.1 Overview of mouse lens development

**FIGURE 1:**
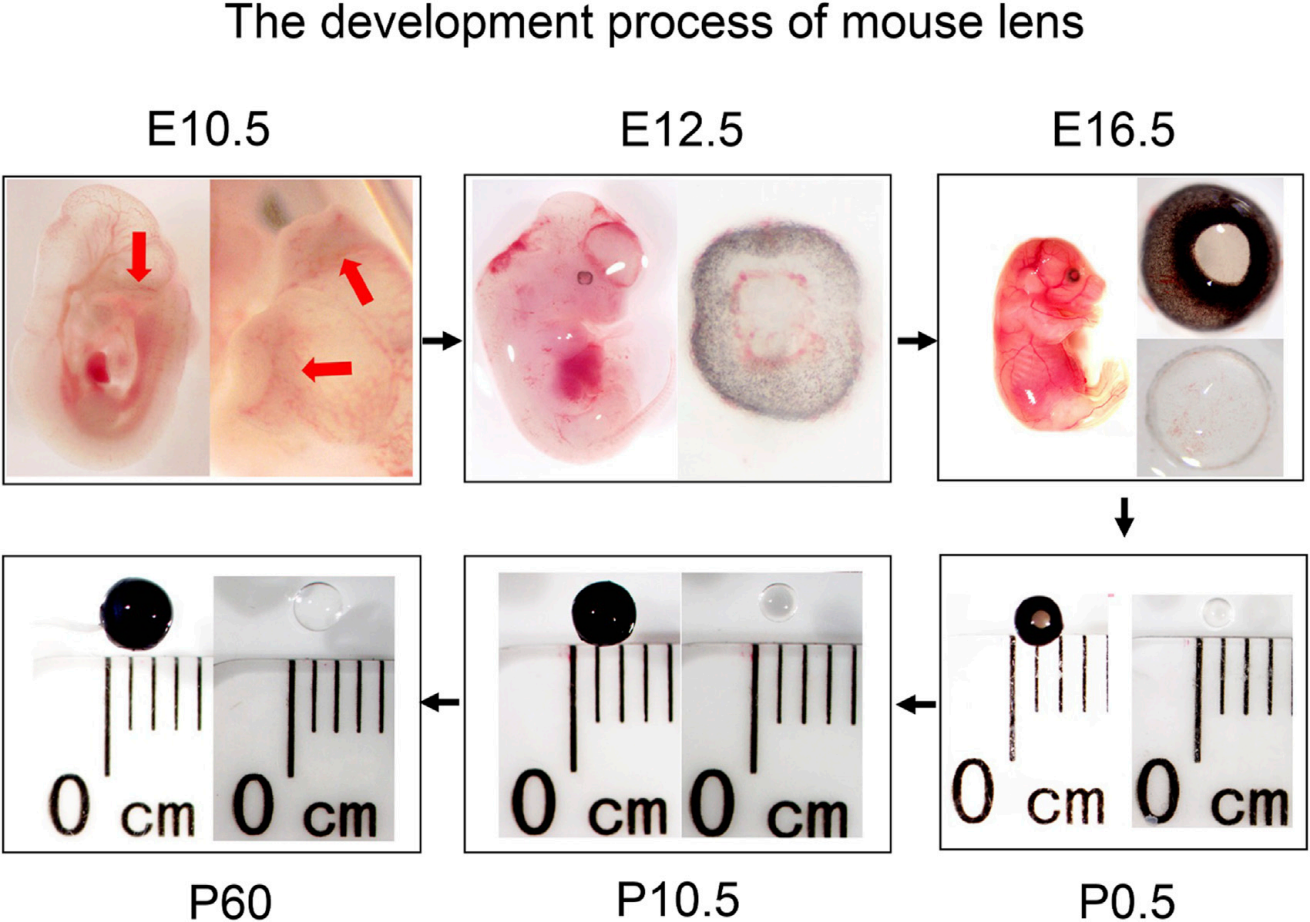
Overview of lens development in mice at different stages (embryonic stages: E10.5, E12.5, and E16.5, and postnatal stages: P0.5, P10.5, and P60). The red arrows indicate the lens pit.

The lens placed invagination to form lens pit and the optic vesicle invaginates to form an optic cup at E10.5. Later, the lens pit is converted into the lens vesicle, and the primary lens fibers began to differentiate, filling the lens vesicle almost completely at E12.5. At E16.5, the secondary lens fiber cells formed and differentiate, and many blood vessels were visible on the lens surface. At neonatal stages (P0.5), the blood vessels on the lens surface decrease and become smooth and round. After birth, as the individual grows and develops, the eyeballs and lens of the mice both gradually become larger (Figure 1).

### 3.2 Summary of the quality of RNA-seq

The quality evaluation of the sequencing data is shown in Table 1. The average clean base number and mapped rate in 18 samples were more than 13.74G and 95.08%, respectively, and the base error rate of more than 90.70% fragments was less than 1‰ (Q30).

**TABLE 1:** Basic sequencing data on six stages of mouse lens development.

Sample	raw_reads	raw_bases (G)	clean_reads	clean_bases (G)	Q30	total_mapped	mapped_rate (%)
E10.5_1	108,066,478	16.21	103,197,594	15.48	91.24	96,980,742	93.98
E10.5_2	95,011,054	14.25	91,012,984	13.65	90.7	85,681,434	94.14
E10.5_3	86,354,354	12.95	85,235,782	12.79	91.73	80,406,226	94.33
E12.5_1	83,330,486	12.5	81,903,970	12.29	91.32	77,058,997	94.08
E12.5_2	84,936,530	12.74	83,708,652	12.56	91.67	80,436,786	96.09
E12.5_3	111,675,766	16.75	109,652,902	16.45	91.32	104,874,534	95.64
E16.5_1	94,320,466	14.15	91,042,866	13.66	91.27	87,359,435	95.95
E16.5_2	95,922,176	14.39	93,142,882	13.97	94.29	89,747,957	96.36
E16.5_3	100,782,342	15.12	97,441,482	14.62	91.68	93,546,868	96.00
P0.5_1	86,613,520	12.99	84,339,492	12.65	94.53	80,698,644	95.68
P0.5_2	92,688,210	13.9	91,185,580	13.68	92.86	86,666,590	95.04
P0.5_3	105,731,364	15.86	102,767,656	15.42	91.5	98,672,941	96.02
P10.5_1	91,317,286	13.7	87,832,442	13.17	94.78	84,709,097	96.44
P10.5_2	95,772,410	14.37	93,856,046	14.08	94.71	90,078,070	95.97
P10.5_3	92,463,608	13.87	90,553,592	13.58	94.42	86,839,679	95.90
P60_1	88,781,608	13.32	86,730,492	13.01	93.56	79,249,183	91.37
P60_2	90,654,686	13.6	88,329,098	13.25	94.25	84,438,108	95.59
P60_3	88,624,054	13.29	86,440,008	12.97	93.92	80,208,694	92.79

### 3.3 Transcriptome analysis of crystalline lens in different developmental stages

**FIGURE 2:**
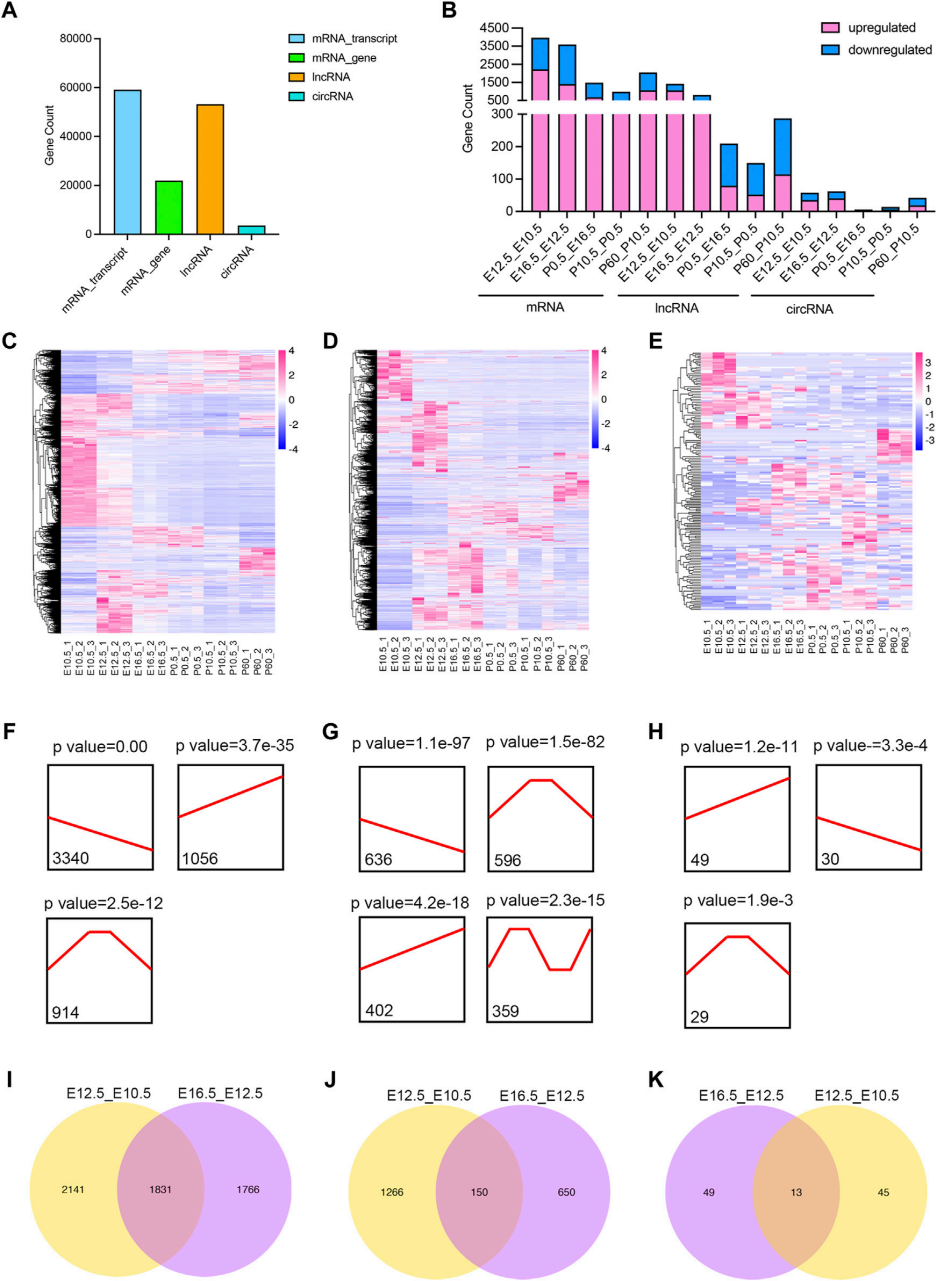
Identification and characteristics of differentially expressed (DE) transcripts in different lens developmental stages (E10.5, E12.5, E16.5, P0.5, P10.5, and P60). **(A)** Counts of mRNAs_gene level, mRNA_transcript level, lncRNAs, and circRNAs in all six stages. **(B)** Counts of DE mRNAs_gene level, DE lncRNAs, and DE circRNAs in five comparison groups (E12.5_E10.5, E16.5_E12.5, P0.5_E16.5, P10.5_P0.5, and P60_P10.5). **(C–E)** Hierarchical clustering analysis of all DE mRNAs **(C)**, DE lncRNAs **(D)**, and DE circRNAs **(E)** among five comparison groups. **(F–H)** Expression trend of DE mRNAs **(F)**, DE lncRNAs **(G)**, and DE circRNAs **(H)** during lens development. Black lines indicate the expression trend. The counts under black lines indicate the number of DE mRNAs, DE lncRNAs, or DE circRNAs with the same expression trend. **(I–K)** Venn plots of co-differentially expressed (CO-DE) mRNAs **(I)**, CO-DE lncRNAs **(J)**, and CO-DE circRNAs **(K)** in E16.5_E12.5 and E12.5_E10.5 groups.

Transcriptional analysis screened a total of 59,145 mRNAs (representing 21,933 genes), 53,195 lncRNAs, and 3,595 circRNAs (Figure 2A; Supplementary Table S2) in six lens developmental stages. Moreover, we identified 3,972, 3,597, 1,484, 979, and 2,042 differentially expressed (DE) mRNAs, 1,416, 800, 209, 149, and 287 DE lncRNAs, and 58, 62, 6, 14, and 42 DE circRNAs in E12.5_E10.5, E16.5_E12.5, P0.5_E16.5, P10.5_P0.5, and P60_P10.5 groups, respectively (Figure 2B; Supplementary Table S3–S5). In summary, a total of 7,123 mRNAs, 2,497 lncRNAs, and 162 circRNAs were identified in all five comparison groups (Figures 2C–E). Moreover, three significantly enriched expression trends, including up-, down-, and up–down, were all observed in mRNAs, lncRNAs, and circRNAs during lens development (Figures 2F–H). Particularly, a significant up–down–up expression trend was only identified in lncRNAs (Figure 2G). Recognizing the critical role of the embryonic development, we performed a comparative analysis of DE genes, identifying 1,831 common differentially expressed (CO-DE) genes, 150 CO-DE lncRNAs, and 13 CO-DE circRNAs shared between the E12.5_E10.5 and E16.5_E12.5 groups (referred to as E16.5_E12.5_E10.5) (Figures 2I–K; Supplementary Table S6).

### 3.4 GO analysis of DE mRNAs and DE circRNAs

**FIGURE 3:**
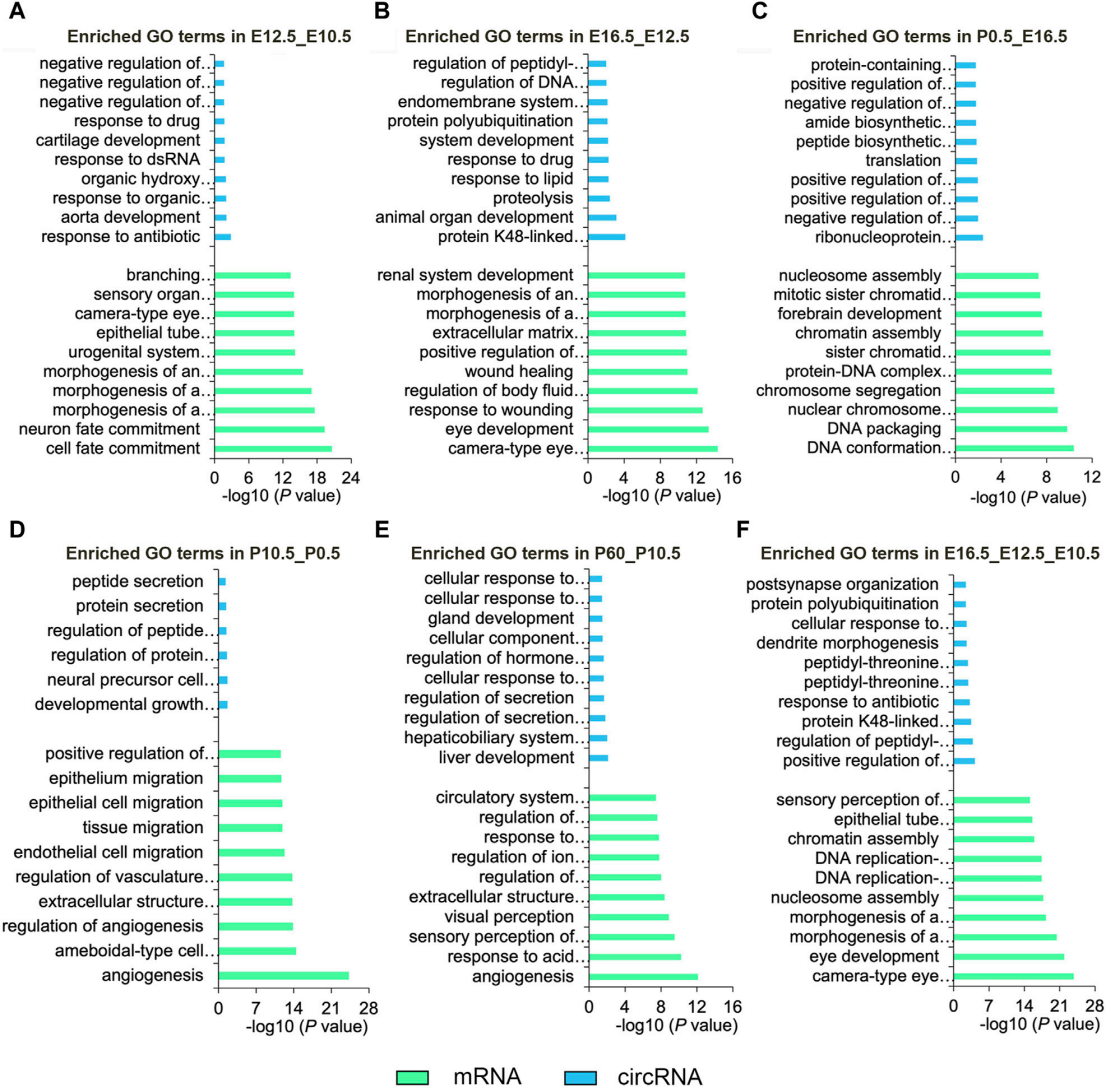
Top 10 enriched biological processes of DE mRNAs and DE circRNAs in six comparison groups (E12.5_E10.5 **(A)**, E16.5_E12.5 **(B)**, P0.5_E16.5 **(C)**, P10.5_P0.5 **(D)**, and P60_P10.5 **(E)**, E16.5_E12.5_E10.5 **(F)**). Blue and green represent circRNAs and mRNAs, respectively.

To clarify the function of the DE mRNAs and circRNAs, we analyzed their main enriched biological processes in the five comparative groups (Figure 3; Supplementary Tables S7, S8). In the E12.5_E10.5 group, DE mRNAs exhibited significant enrichment in cell fate commitment and epithelial morphogenesis, while DE circRNAs were enriched in response to cyclic compounds and negative regulation of biosynthetic processes (Figure 3A). In the E16.5_E12.5 group, DE mRNAs were notably associated with camera-type eye development and epithelial morphogenesis, while DE circRNAs showed enrichment in animal organ development and protein hydrolysis (Figure 3B). In the P0.5_E16.5 group, DE mRNAs were enriched in DNA conformational changes and chromosome segregation, while DE circRNAs were linked to the regulation of RNA metabolism and apoptotic processes (Figure 3C). In the P10.5_P0.5 group, significantly enriched GO terms were mainly associated with angiogenesis and epithelial migration for DE mRNAs and morphogenesis and regulation of protein secretion for DE circRNAs (Figure 3D). In the P60_P10.5 group, the significantly enriched GO terms were mainly enriched in angiogenesis and visual perception of DE mRNAs, regulation of secretion, and cell response to oxidative stress of DE circRNAs (Figure 3E). Furthermore, we analyzed the biological processes involving the CO-DE mRNAs and CO-DE circRNAs in E16.5_E12.5_E10.5. Our findings revealed that CO-DE mRNAs were predominantly enriched in camera-type eye development and epithelial tube morphogenesis, while CO-DE circRNAs were linked to the regulation of peptidyl-threonine phosphorylation and dendrite morphogenesis (Figure 3F; Supplementary Table S5).

### 3.5 GO analysis of DE lncRNAs

**FIGURE 4:**
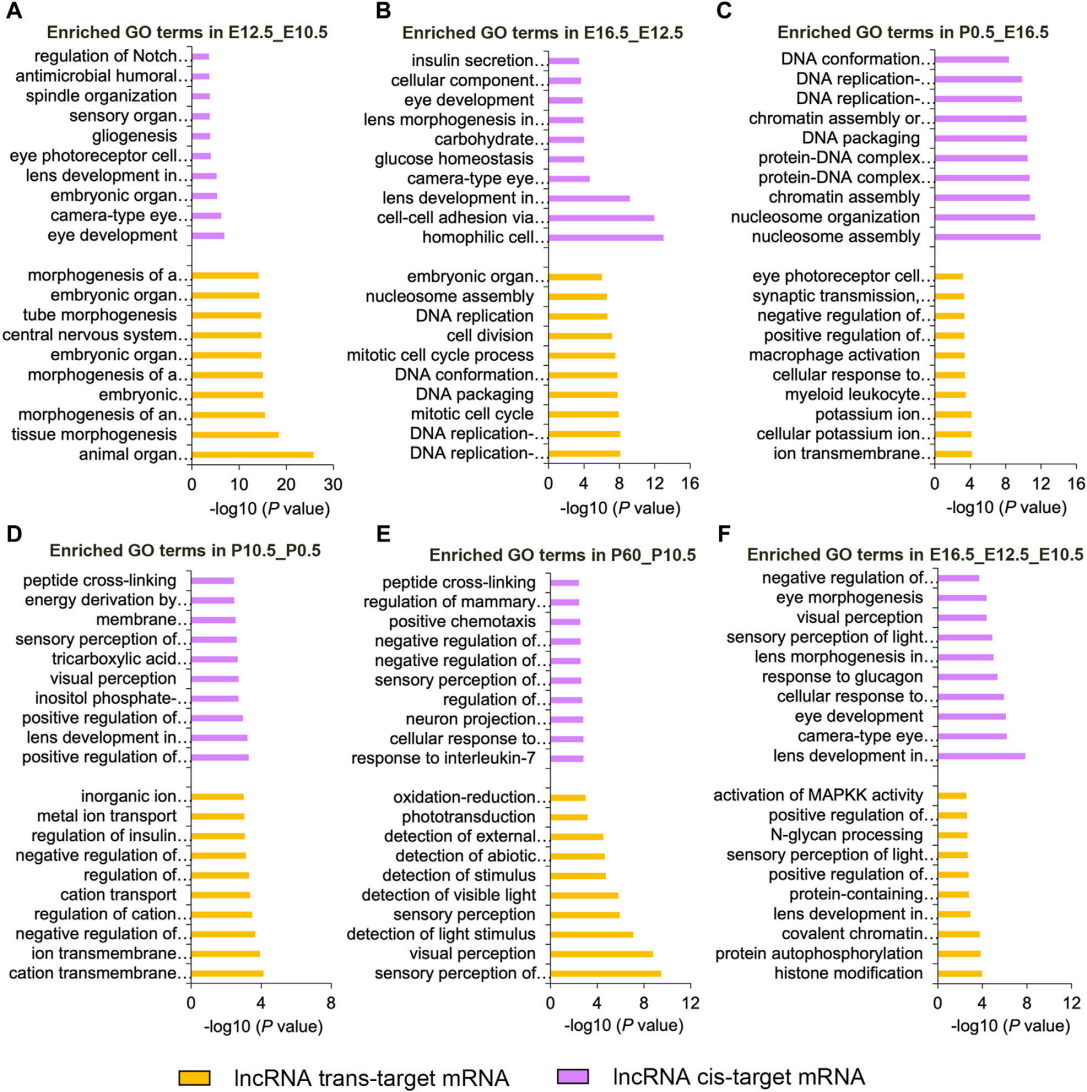
Top 10 enriched biological processes of DE lncRNAs cis- or trans-targeted mRNAs in six comparison groups (E12.5_E10.5 **(A)**, E16.5_E12.5 **(B)**, P0.5_E16.5 **(C)**, P10.5_P0.5 **(D)**, and P60_P10.5 **(E)**, E16.5_E12.5_E10.5 **(F)**). Purple and yellow represent cis-targeted and trans-targeted mRNAs, respectively.

Based on the cis-targeting (co-localization) and trans-targeting (co-expression) assays, we further investigated the functions of DE lncRNAs in each comparison group by analyzing their target genes (Figure 4; Supplementary Table S9, S10). In the E12.5_E10.5 group, significantly enriched GO terms were mainly related to organ morphogenesis and camera-type eye development for DE lncRNA cis-targeted genes and embryonic organ morphogenesis and morphogenesis of an epithelium for DE lncRNA trans-targeted genes (Figure 4A). In the E16.5_E12.5 group, significant enriched GO terms were primarily involved in cell–cell adhesion via plasma-membrane adhesion molecules and camera-type eye development for DE lncRNA cis-targeted genes and DNA conformation changes and embryonic organ morphogenesis for DE lncRNA trans-targeted genes (Figure 4B). In the P0.5_E16.5 group, significant enriched GO terms were primarily related to the chromatin assembly and protein-DNA complex assembly for DE lncRNA cis-targeted genes and ion transmembrane transport and eye photoreceptor cell development for DE lncRNA trans-targeted genes (Figure 4C). In the P10.5_P0.5 group, significantly enriched GO terms were mainly enriched in the positive regulation of RNA splicing and lens development in camera-type eyes for DE lncRNA cis-targeted genes and ion transmembrane transport and regulation of insulin secretion for DE lncRNA trans-targeted genes (Figure 4D). In the P60_P10.5 group, significant enriched GO terms were closely relevant to interleukin-7 and sensory perception of light stimulus for DE lncRNA cis-targeted genes and sensory perception of light stimulus and phototransduction for DE lncRNA trans-targeted genes (Figure 4E). In addition, we analyzed the biological processes involving the CO_DE lncRNAs in the E16.5_E12.5_E10.5 group. Cis-targeted analysis identified significantly enriched biological processes for the CO_DE lncRNAs, such as lens development in camera-type eyes and visual perception. Moreover, trans-targeted analysis showed that DE lncRNAs were mainly involved in histone modification and lens development in camera-type eyes (Figure 4F).

### 3.6 GO analysis of significantly enriched dynamical trends of mRNAs, lncRNAs, and circRNAs

**FIGURE 5:**
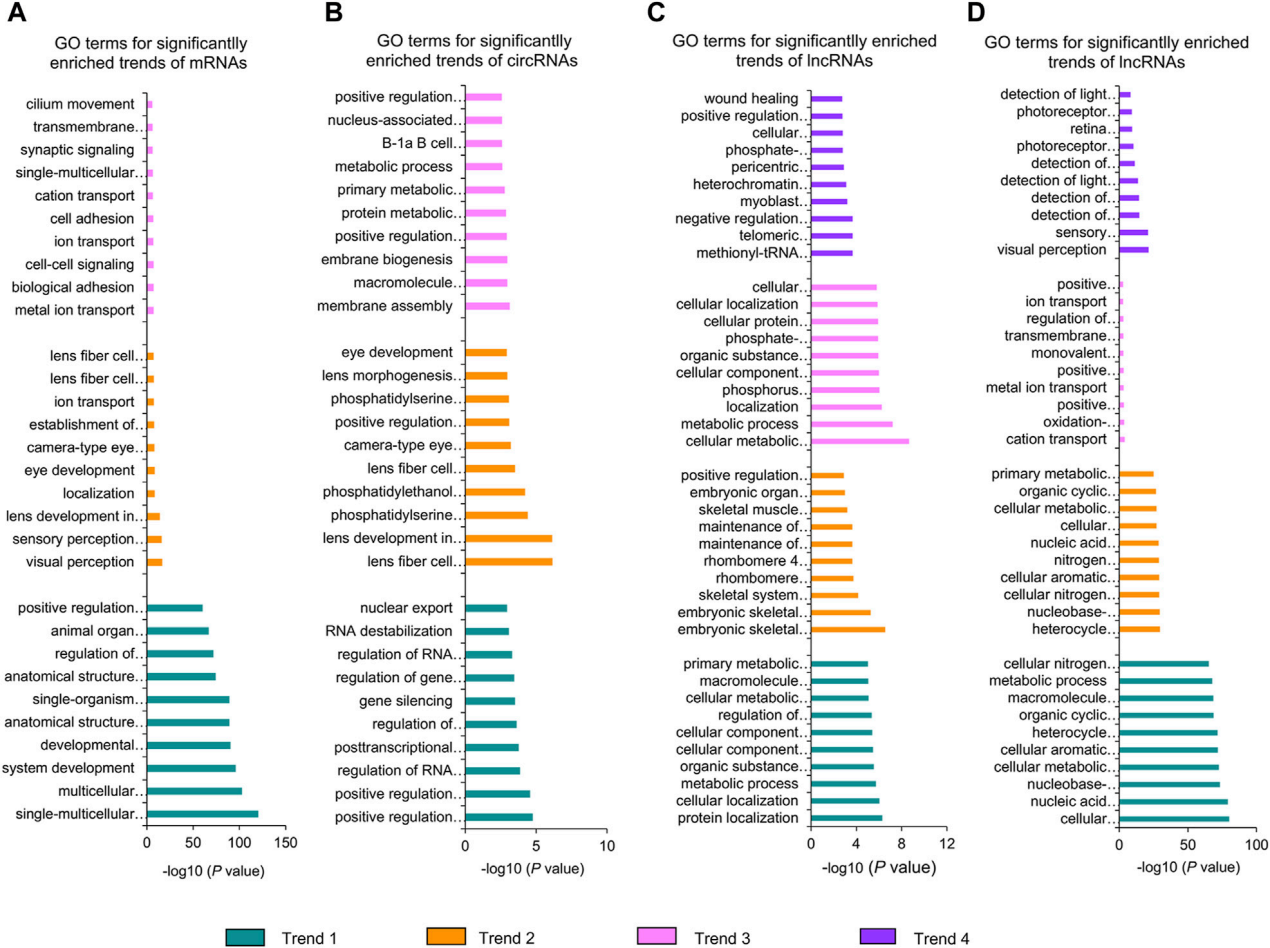
GO analysis of significantly enriched dynamical trends of mRNAs **(A)**, circRNAs **(B)**, lncRNA cis-target genes **(C)**, and lncRNA trans-target genes **(D)**. Trend 1, trend 2, trend 3, and trend 4 represent downregulated, upregulated, up–down-regulated, and up–down–up-regulated dynamical trends in the whole stage of lens development, respectively.

In addition, we conducted GO analysis on the significantly enriched dynamical trends observed in Figures 2F–H (Figure 5; Supplementary Table S11). During the whole stage of lens development, the downregulated mRNAs were mainly enriched in the developmental process and anatomical structure morphogenesis, the upregulated mRNAs were closely relevant to lens development in camera-type eye and ion transport, the up–down-regulated mRNAs were mainly associated with metal ion transport and biological adhesion (Figure 5A). The downregulated circRNAs were mainly enriched in the regulation of RNA export from the nucleus and regulation of gene expression, epigenetic; the upregulated circRNAs were primarily related to lens development in camera-type eye and phosphatidylserine metabolic processes; and the up-down-regulated circRNAs were mainly associated with membrane biogenesis and macromolecule metabolic processes (Figure 5B). Moreover, downregulated lncRNA cis-target genes were mainly enriched in cellular localization and the metabolic process, the upregulated lncRNAs cis-target mRNAs were mainly associated with embryonic skeletal system morphogenesis and embryonic organ morphogenesis, the up–down-regulated lncRNAs cis-target mRNAs were mainly related to the metabolic process and cellular component biogenesis, and the up–down–up-regulated lncRNAs cis-target mRNAs were mainly involved in methionyl-tRNA aminoacylation and the telomeric heterochromatin assembly (Figure 5C). The downregulated or upregulated lncRNAs trans-target mRNAs were both mainly enriched in the cellular metabolic process, the up–down-regulated lncRNAs trans-target mRNAs were mainly associated with cation transport and oxidation–reduction process, and the up–down–up-regulated lncRNAs trans-target mRNAs were primarily relevant to visual perception and photoreceptor cell development (Figure 5D).

### 3.7 Heat map clusters and protein–protein interaction (PPI) network construction of DE genes related to eye development

**FIGURE 6:**
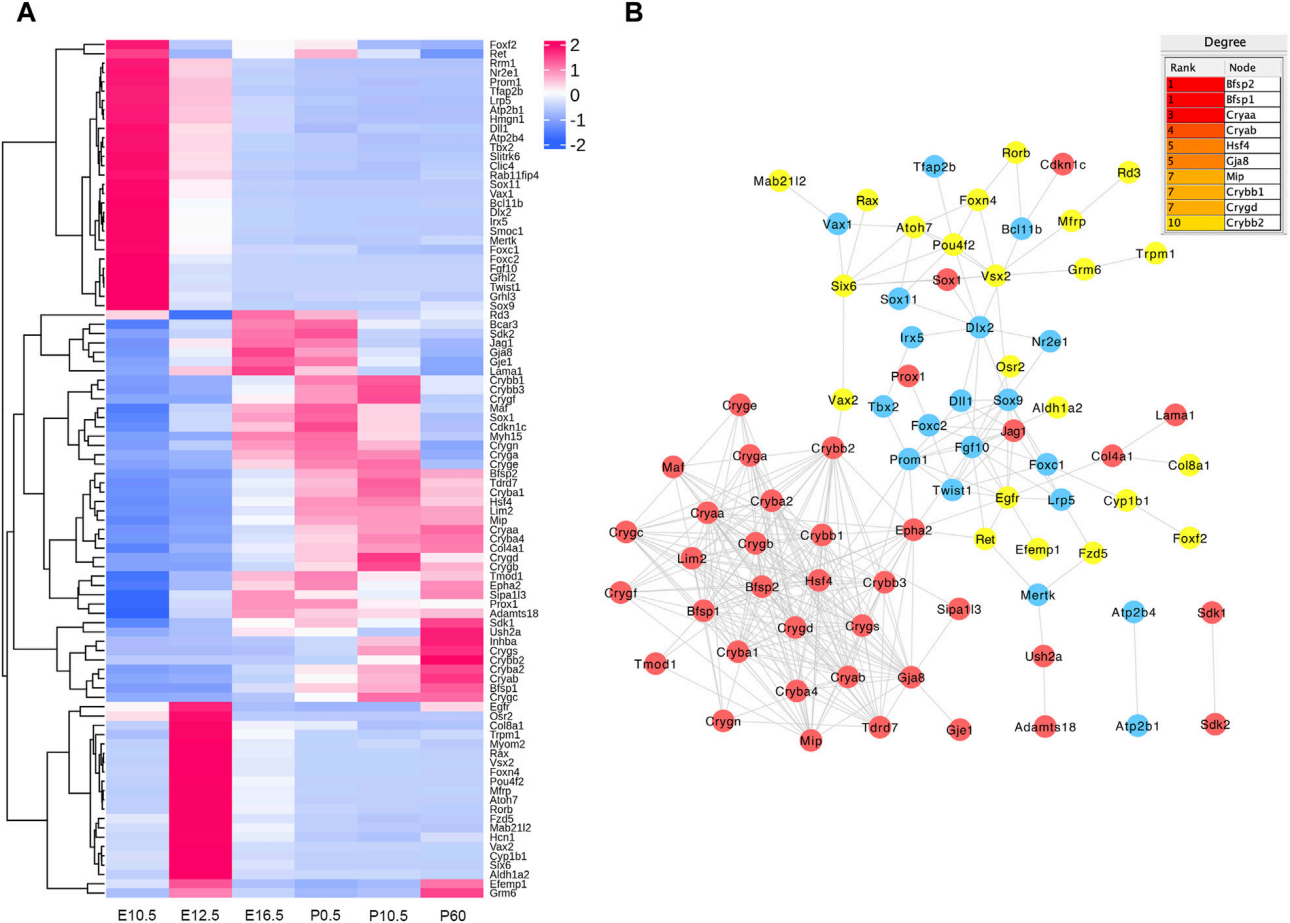
Hierarchical clustering analysis **(A)** and protein–protein interaction network **(B)** of co-differentially expressed (CO-DE) mRNAs in the E16.5_E12.5_E10.5 group associated with eye development. Red, blue, and yellow represent both upregulated, both downregulated, and inconsistent trends in E12.5_E10.5 and E16.5_E12.5, respectively.

**FIGURE 7:**
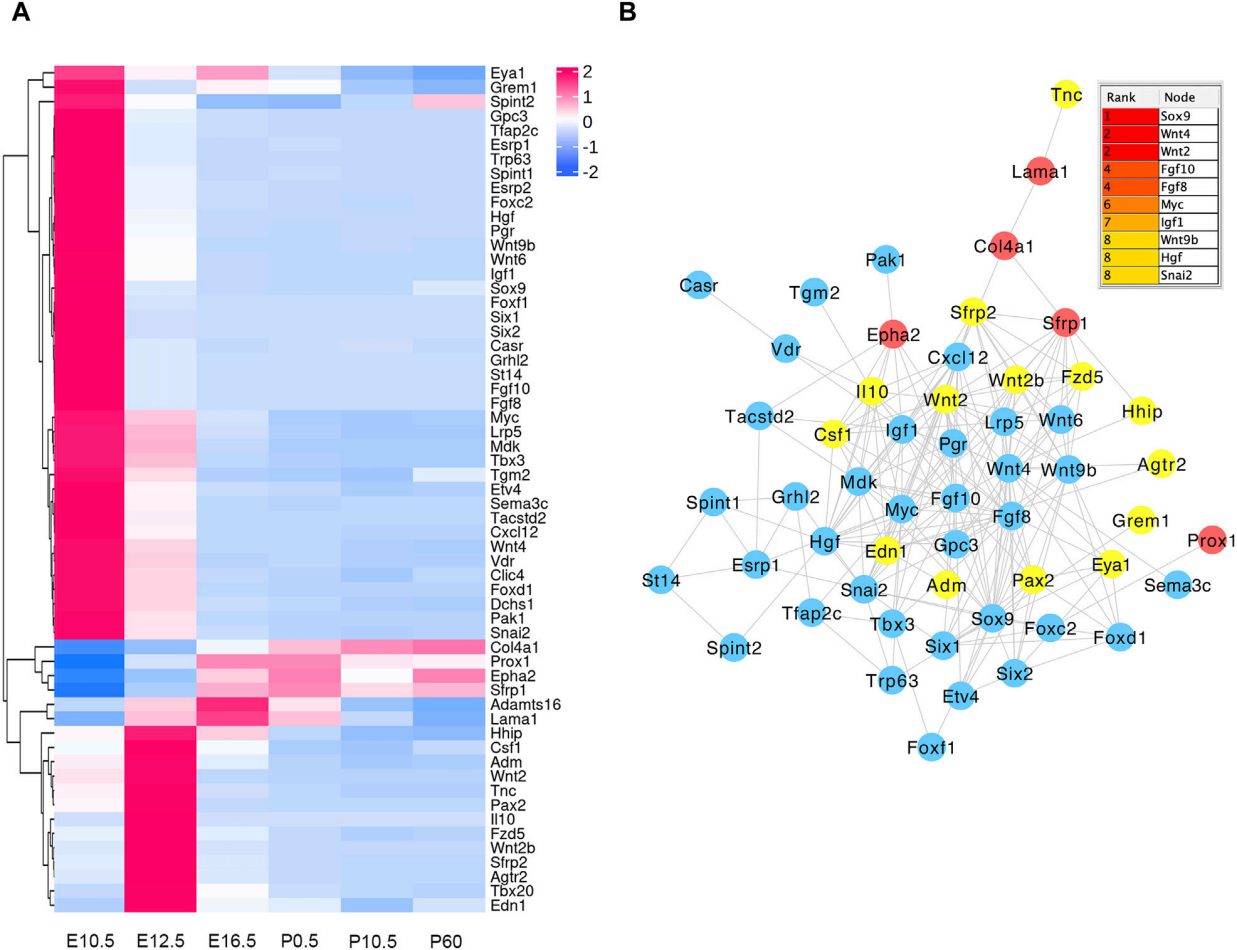
Hierarchical clustering analysis **(A)** and protein–protein interaction network **(B)** of co-differentially expressed (CO-DE) mRNAs in the E16.5_E12.5_E10.5 group associated with the morphogenesis of a branching epithelium. Red, blue, and yellow represent both upregulated, both downregulated, and inconsistent trends in E12.5_E10.5 and E16.5_E12.5 groups, respectively.

In the E16.5_E12.5_E10.5 group, we identified 92 CO-DE mRNAs associated with eye development and 59 CO-DE mRNAs linked to the morphogenesis of a branching epithelium. As depicted in Figure 6A, the majority of genes implicated in eye development exhibited lower expression levels at E10.5 and E12.5. Among them, 79 genes demonstrated interaction relationships within the protein–protein interaction (PPI) network, with the top 10 hub genes, crucial for lens development, identified through the cytoHubba plugin in Cytoscape software using the degree method. These hub genes include *BFSP2*, *BFSP1*, five crystalline proteins (*CRYAA*, *CRYAB*, *CRYBB1*, *CRYGD*, and *CRYBB2*), *HSF4*, *GJA8*, and *MIP* (Figure 6B)*.* Conversely, the mRNAs associated with the morphogenesis of a branching epithelium displayed higher expression levels at E10.5 and E12.5, as illustrated in Figure 7A. Among these genes, 54 genes demonstrated interaction relationships in the PPI network (Figure 7B). Employing the same analytical approach, we identified the top 10 hub genes crucial for lens development, including *SOX9*, *WNT4*, *WNT2*, *FGF10*, *FGF8*, *MYC*, *IGF1*, *WNT9b*, *HGF*, and *SNAI2* (Figure 7B).

### 3.8 Regulatory networks of CO-DE lncRNAs and target mRNAs

**FIGURE 8:**
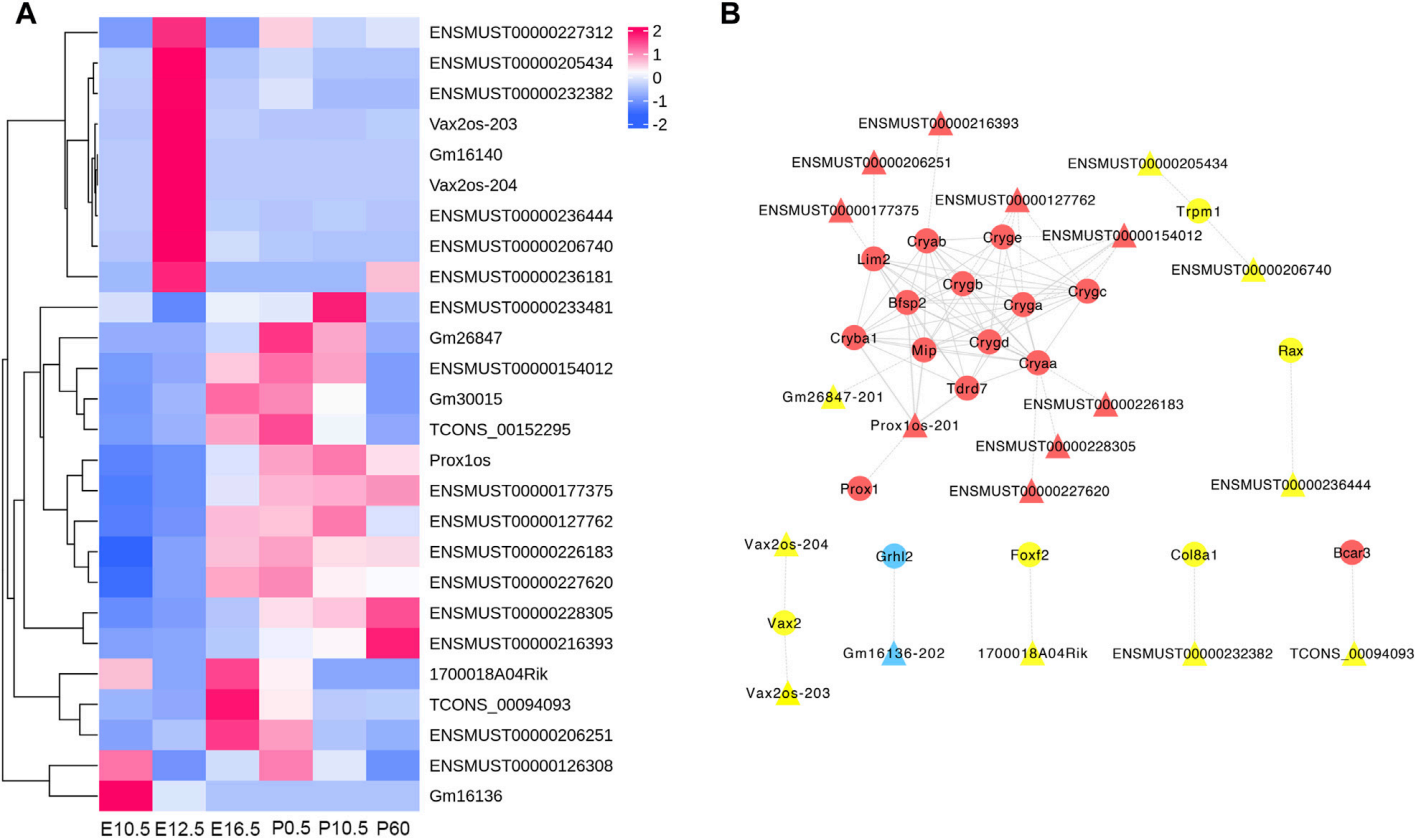
Regulatory network of codifferentially expressed (CO-DE) lncRNAs in E16.5_E12.5 and E12.5_E10.5 groups **(A)** and regulatory network of CO-DE lncRNAs and their CO-DE cis- or trans-targeted mRNAs associated with lens development **(B)**. Circles and triangles represent mRNAs and lncRNAs, respectively. Red, blue, and yellow represent both upregulated, both downregulated, and inconsistent trends in E12.5_E10.5 and E16.5_E12.5 groups, respectively. Dashed, solid, and contiguous arrow lines represent cis-target, trans-target, and PPI interaction relationships, respectively.

To better reflect the interactive relationship between lncRNAs and mRNAs, we structured a regulatory network on the basis of these CO-DE lncRNAs and their target genes related to the eye development process. The regulatory networks included 19 CO-DE lncRNAs and 20 CO-DE genes, forming 86 interaction relationships, including 27 cis-targeted relationships, 5 trans-targeted relationships, and 54 protein–protein interaction relationships (Figure 8; Supplementary Table S12).

### 3.9 Construction of the circRNA–miRNA–mRNA regulatory network

**FIGURE 9:**
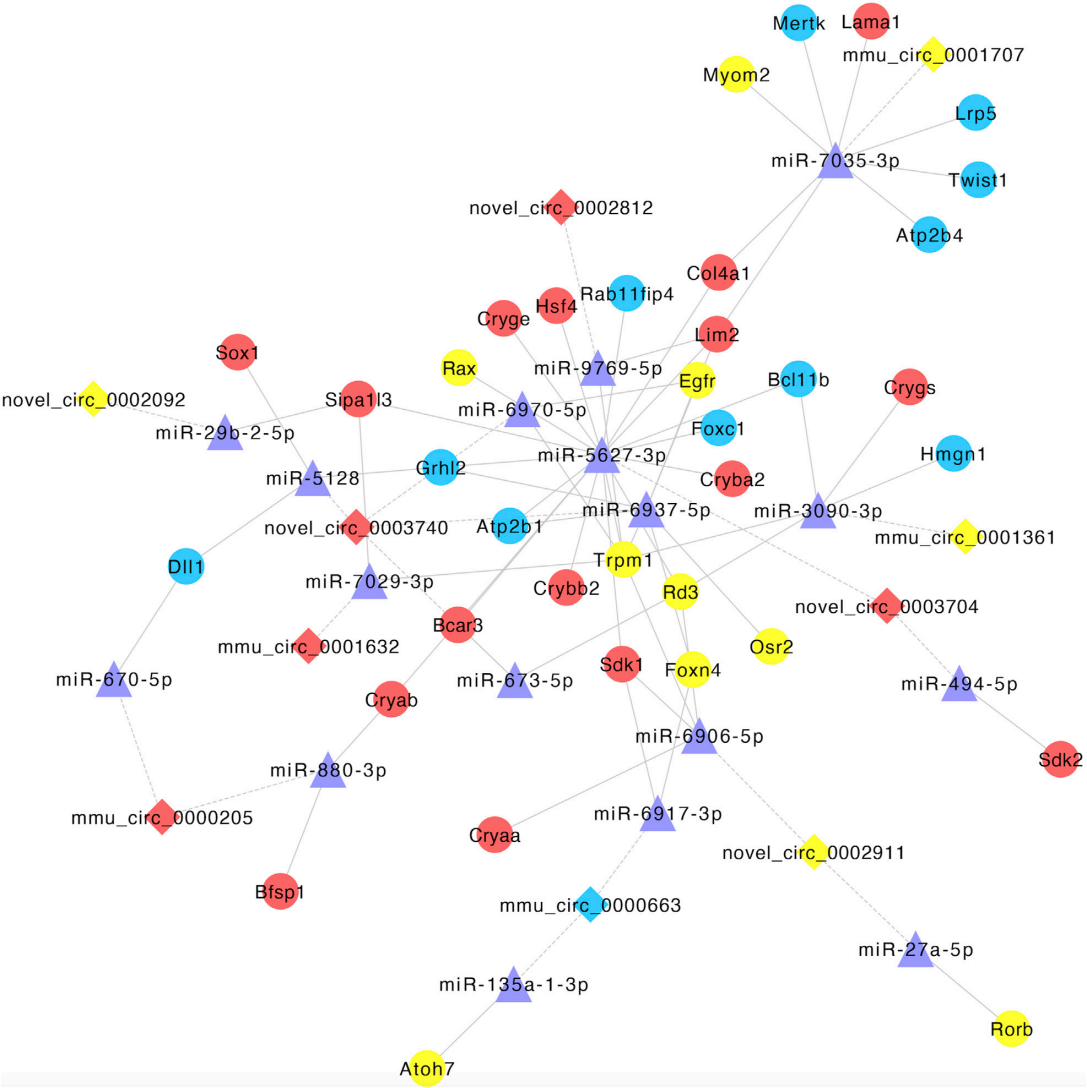
Establishment of the circRNA–miRNA–mRNA competitive endogenous RNA network. Diamonds and circles represent circRNA and mRNA, respectively. Purple triangles represent miRNA. Red, blue, and yellow represent both upregulated, both downregulated, and inconsistent trends in E12.5_E10.5 and E16.5_E12.5 groups, respectively. The solid lines represent miRNA–mRNA interactions, and dotted lines represent miRNA–circRNA interactions.

Based on the relationships between CO-DE circRNAs and mRNAs and the role of miRNA as a middle regulatory molecule, we obtained the pairs of miRNA–mRNA and miRNA–circRNA. In order to screen miRNAs and circRNAs related to lens development, we selected the co-expression relationships, which involved in genes related to lens development, and constructed a circRNA–miRNA–mRNA ceRNA network. The co-expression network was composed of 10 circRNAs, 17 miRNAs, and 36 mRNAs (Figure 9; Supplementary Table S13).

### 3.10 Validation of DE genes by qRT-PCR

**FIGURE 10:**
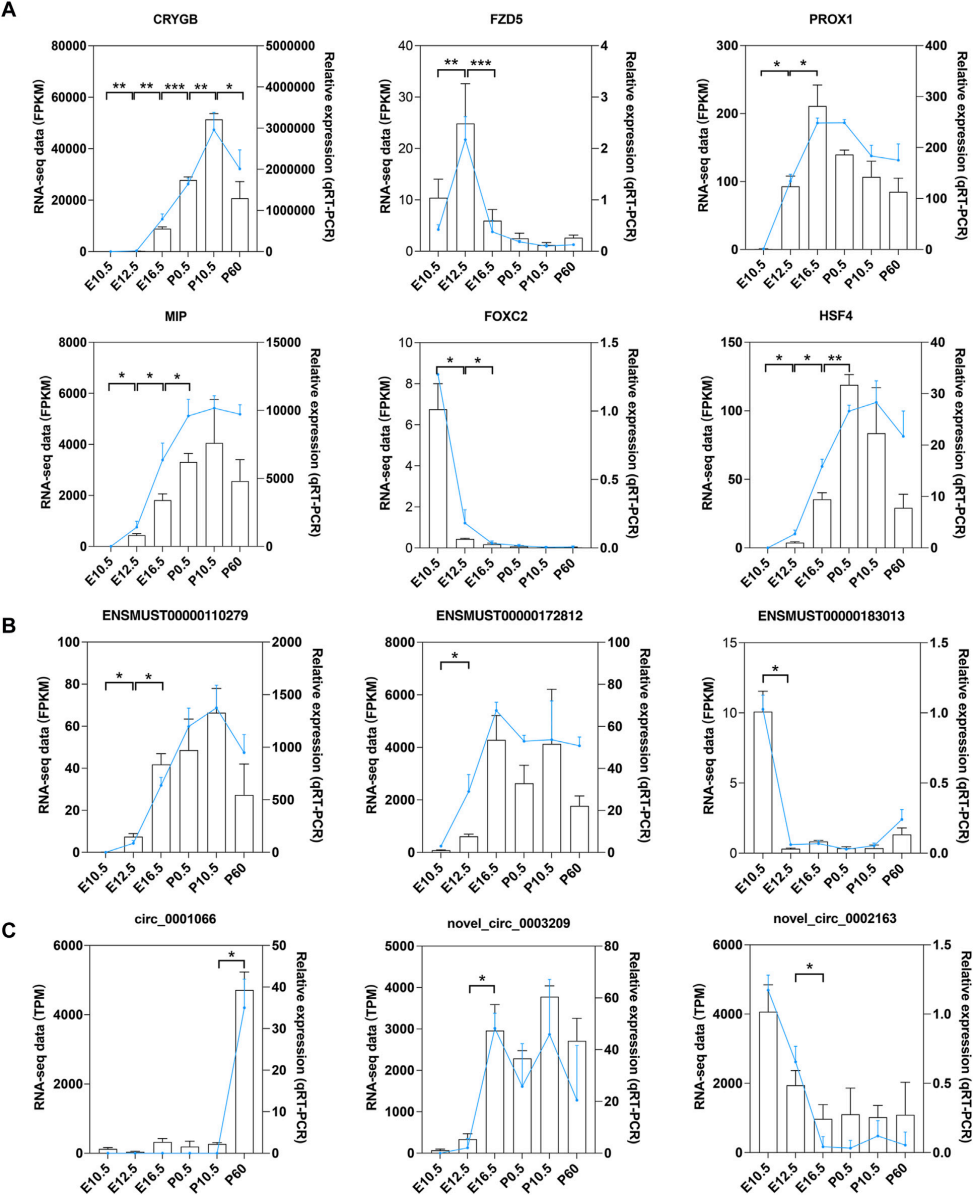
qRT-PCR validation of mRNAs, lncRNAs, and circRNAs. qRT-PCR validation of the expression levels in six stages of mouse lens development for **(A)** six DE mRNAs, **(B)** three DE lncRNAs, and **(C)** three DE circRNAs. Data from RNA-seq are shown as lines labeled on the Y-axis on the left. Data from qRT-PCR are shown as columns labeled on the Y-axis on the right; *p < 0.05, **p < 0.01, and ***p < 0.001.

A total of 12 genes, including 6 mRNAs (*CRYGB*, *MIP*, *PROX1*, *FZD5*, *FOXC2*, and *HSF4*), 3 lncRNAs (*ENSMUST00000110279*, *ENSMUST00000172812*, and *ENSMUST00000183013*), and 3 circRNAs (*circ_0001066*, *novel_circ_0002163*, and *novel_circ_0003209*), were selected for qRT-PCR verification. Results showed that the expression patterns of these genes in qRT-PCR were consistent with the data from RNA-seq, indicating that the results of RNA-seq are reliable (Figure 10).

## 4 Discussion

Embryonic lens formation is a critical step in eye organ development, and the continuous division and differentiation of epithelial cells at the equator forms the human lens. Despite this constant remodeling process occurring within a confined environment, the lens maintains its transparency through the intricate regulation of cell signaling pathways within the lens epithelial cells (Bron et al., 2000; Danysh and Duncan, 2009). In this study, we profiled mRNAs, lncRNAs, and circRNAs in the developing mouse lens and focused on key ncRNAs and mRNAs related to lens development.

A total of 59,145 mRNAs (21,933 genes), 53,195 lncRNAs, and 3,595 circRNAs were identified in six lens developmental stages. A previous study probed 9,733 mRNAs and 1,952 long intergenic ncRNA in the developing mouse lens (Khan et al., 2016; Anand et al., 2018). The most differentially expressed (DE) mRNAs, lncRNAs, and circRNAs belonged to the E12.5_E10.5 and E16.5_E12.5 groups. GO enrichment analysis indicated that DE mRNAs and lncRNAs in the embryonic period were mainly associated with lens development, and DE mRNAs in the postnatal period were mainly associated with angiogenesis. During mammalian embryonic development, the lens is enveloped by a capillary network from the vitreous vascular system, playing a crucial role in lens development and maturation (Ito and Yoshioka, 1999). The lens vasculature in mice begins to degenerate after birth and is completely regressed at about 3 weeks, consistent with the observed phenotype of abundant blood vessels covering the lens at E16.5 (Jack, 1972). The degeneration process of lens involves the orderly contraction of the lumen and apoptosis of endothelial cells (Latker and Kuwabara, 1981; Ito and Yoshioka, 1999; Zhu et al., 2000). Vascular endothelial growth factor A (*VEGFA*) is expressed at an early stage of lens development and is essential for stimulating vascular endothelial cell proliferation (Ash and Overbeek, 2000; Shui et al., 2003). Deletion of VEGFA can lead to reduced vascular membrane production, resulting in smaller lenses with mild nuclear turbidity after birth, while overexpression can lead to excessive capillary formation and endothelial cell proliferation and deposition (Ash and Overbeek, 2000; Mitchell et al., 2006; Garcia et al., 2009). Ocular ischemia or inflammation may lead to the entry of VEGF into the lens capsule, resulting in the formation of new blood vessels (Roy et al., 2020).

Our data revealed that key hub genes related to eye development exhibited lower expression levels during the embryonic stage. Mutations in these genes, such as beaded filament structural proteins 1 and 2 (*BFSP1* and *BFSP2*) and lens-specific connexin proteins (*GJE1* and *GJA8*), have been linked to cataract formation (Song et al., 2009; Beyer et al., 2013). Additionally, the heat shock factor 4 (HSF4) and major intrinsic protein (MIP) play crucial roles in lens cell growth, differentiation, and maintaining optical quality (Fujimoto et al., 2004; Bennett et al., 2016).

Conversely, the top 10 enriched hub genes related to the morphogenesis of a branching epithelium showed higher expression levels during the embryonic stage. The SOX proteins, characterized by a highly conserved DNA-binding and bending domain known as an HMG box, play a crucial role in regulating lacrimal gland branching and differentiation (Chen et al., 2014). SRY-box transcription factor 9 (*SOX9*) was initially expressed in the lens pit, and Dct-Sox9 transgenic mice reported are accompanied with microphthalmia with cataract (Qin et al., 2004; Chen et al., 2014). Moreover, Wnt signaling and growth factors are also involved in lens development, which are reported to be necessary for inducing lens formation and ectodermal differentiation (Chen et al., 2004; Lovicu and McAvoy, 2005). Previous studies have shown that during the development of the lens during the embryonic stage, the expression level of *MYC* (MYC proto-oncogene, BHLH transcription factor) and *MDK* (Midkine) gradually decreases, which is consistent with our results (Cavalheiro et al., 2014). *MYC* can regulate cell proliferation during lens development, and its deletion can lead to severe lens developmental defects (Cavalheiro et al., 2014; Cui and Lwigale, 2019). In addition, the expression level of SNAI family transcriptional repressor 2 (SNAI2) was highly upregulated in lens epithelial cells obtained from patients with anterior polar cataracts, indicating that it may involve in lens development (Choi et al., 2007). At present, there is very limited research on *SOX9*, *MDK,* and *SNAI2* in lens development, and more work is needed to further clarify their functions.

To excavate the key potential lncRNAs involved in lens development, we obtained CO-DE lncRNAs, which are cis- or trans-targeted CO-DE mRNAs related to lens development, and constructed an mRNA–lncRNA co-expression network. Several identified lncRNAs have been previously implicated in eye development studies. For example, lncRNA Vax2os were cis-targeted with ventral anterior homeobox 2 (*VAX2*), and were highly expressed at E12.5. Previous studies have validated the function of Vax2os, which is involved in neurotransmitter transport during mouse retinal development and plays an important role in regulating eye development (Alfano et al., 2005; Chen et al., 2021). LncRNAs TCONS_00094093 were cis-targeted with BCAR3 adaptor protein (*BCAR3*), the signaling mediated by which is required for the maintenance of the structural integrity of the ocular lens (Near et al., 2009; Kondo et al., 2016). LncRNAs Prox1os were cis-targeted with Prospero Homeobox 1 (*PROX1*) and trans-target with crystallin beta A1 (*CRYBA1*) and *BFSP2*, which has been showed is crucial for mouse lens fiber differentiation and elongation (Wigle et al., 1999; Song et al., 2009; Audette et al., 2016; Joseph et al., 2023).

Research has shown that mRNAs, lncRNAs, and circRNAs regulate each other’s expression by acting as competitive endogenous RNA (miRNA sponges). Utilizing the CO-DE mRNAs and lncRNAs, along with predicted targeted miRNAs, we constructed a competitive endogenous RNA (ceRNA) network to elucidate the key factors potentially involved in lens development during embryogenesis. For instance, miR-135a, was co-expressed with atonal bHLH transcription factor 7 (*ATOH7*) and circ_0000663. miR-135a was upregulated in the aqueous humor of patients with cataract and primary open-angle glaucoma and may be involved in the regulation of the pathogenesis of anterior segment diseases (Dunmire et al., 2013; Wang et al., 2023). The *ATOH7* gene encodes a transcription factor involved in determining the fate of retinal progenitor cells and is necessary for the development of the optic nerve, and mutations in this gene have been reported to cause severe eye developmental defects (Khan et al., 2012). Moreover, miR-29b was co-expressed with novel_circ_0002092 and signal-induced proliferation-associated 1-like 3 (*SIPA1L3*). Research has shown that miR-29b is involved in the pathogenesis of myotonic dystrophy type 1 cataract and can regulate the function of lens epithelial cells by affecting the Ca^2+^ concentration (Shao et al., 2017; Gao et al., 2021). *SIPA1L3* encodes a putative GTPase-activating protein, which was necessary for lens and eye development (Greenlees et al., 2015). Thus, these RNAs contained in the ceRNA network may play important roles in eye development, and further functional verification is necessary.

We recognize there are some limitations to this study. First, due to the limited amount of lens tissue in embryonic mice, we did not perform miRNA profiling analysis. Second, due to the inability to separate the lens epithelium and lens capsule during the early stages of lens development, we did not sequence them separately. Third, this study mainly focused on mRNA and non-coding RNA expression profiles during lens development and lacked functional experiments on the molecular mechanisms of non-coding RNAs, which should be further explored in future studies.

In conclusion, our data provide a comprehensive analysis of mRNAs, lncRNAs, and circRNAs and biological processes that involved in lens development from embryonic to postnatal stages. However, the mechanisms and biological functions of lncRNAs and circRNAs in eye development remain to be further explored.

## Data Availability

The datasets presented in this study can be found in online repositories. The names of the repository/repositories and accession number(s) can be found in the article/Supplementary Material.

## Supplementary material

SUPPLEMENTARY TABLE S1 Primer sequences used for quantitative real-time PCR validation.

SUPPLEMENTARY TABLE S2 Genes expressed in six developmental stages of mice lens (E10.5, E12.5, E16.5, P0.5, P10.5, and P60).

SUPPLEMENTARY TABLE S3 Differentially expressed mRNAs in five comparison groups (E12.5_E10.5, E16.5_E12.5, P0.5_E16.5, P10.5_P0.5, and P60_P10.5).

SUPPLEMENTARY TABLE S4 Differentially expressed lncRNAs in five comparison groups (E12.5_E10.5, E16.5_E12.5, P0.5_E16.5, P10.5_P0.5, and P60_P10.5).

SUPPLEMENTARY TABLE S5 Differentially expressed circRNAs in five comparison groups (E12.5_E10.5, E16.5_E12.5, P0.5_E16.5, P10.5_P0.5, and P60_P10.5).

SUPPLEMENTARY TABLE S6 Common differentially expressed genes in the E16.5_E12.5_E10.5 group.

SUPPLEMENTARY TABLE S7 GO terms for differentially expressed mRNAs in six comparison groups (E12.5_E10.5, E16.5_E12.5, P0.5_E16.5, P10.5_P0.5, P60_P10.5, and E16.5_E12.5_E10.5).

SUPPLEMENTARY TABLE S8 GO terms for differentially expressed circRNAs in six comparison groups (E12.5_E10.5, E16.5_E12.5, P0.5_E16.5, P10.5_P0.5, P60_P10.5, and E16.5_E12.5_E10.5).

SUPPLEMENTARY TABLE S9 GO terms for differentially expressed lncRNAs cis-targeted mRNAs in six comparison groups (E12.5_E10.5, E16.5_E12.5, P0.5_E16.5, P10.5_P0.5, P60_P10.5, and E16.5_E12.5_E10.5).

SUPPLEMENTARY TABLE S10 GO terms for differentially expressed lncRNAs trans-targeted mRNAs in six comparison groups (E12.5_E10.5, E16.5_E12.5, P0.5_E16.5, P10.5_P0.5, P60_P10.5, and E16.5_E12.5_E10.5).

SUPPLEMENTARY TABLE S11 GO terms for significantly enriched dynamical trends of mRNAs, circRNAs, and lncRNAs.

SUPPLEMENTARY TABLE S12 List of mRNA–lncRNA interaction.

SUPPLEMENTARY TABLE S13 List of circRNA–miRNA and miRNA–mRNA interaction.
